# Head motion classification using thread-based sensor and machine learning algorithm

**DOI:** 10.1038/s41598-021-81284-7

**Published:** 2021-01-29

**Authors:** Yiwen Jiang, Aydin Sadeqi, Eric L. Miller, Sameer Sonkusale

**Affiliations:** 1 Department of Electrical and Computer Engineering, Tufts University, 161 College Ave, Medford, MA 02155 USA; 2 Nano Lab, Advanced Technology Laboratory, Tufts University, 200 Boston Ave, Medford, MA 02155 USA

**Keywords:** Electrical and electronic engineering, Computer science

## Abstract

Human machine interfaces that can track head motion will result in advances in physical rehabilitation, improved augmented reality/virtual reality systems, and aid in the study of human behavior. This paper presents a head position monitoring and classification system using thin flexible strain sensing threads placed on the neck of an individual. A wireless circuit module consisting of impedance readout circuitry and a Bluetooth module records and transmits strain information to a computer. A data processing algorithm for motion recognition provides near real-time quantification of head position. Incoming data is filtered, normalized and divided into data segments. A set of features is extracted from each data segment and employed as input to nine classifiers including Support Vector Machine, Naive Bayes and KNN for position prediction. A testing accuracy of around 92% was achieved for a set of nine head orientations. Results indicate that this human machine interface platform is accurate, flexible, easy to use, and cost effective.

## Introduction

The monitoring of head motions can be very useful in many applications including the study of human behavior. For example, it can improve our understanding of one’s attention, focus and emotional state. In a classroom setting, head movements can be translated into students’ gaze direction and serve to detect students’ attentiveness during class^1^. Similarly, this technology can be applied to a driving situation, where a distracted or tired driver can be alerted through detecting abnormalities in their head motion. Head motions such as tilting and nodding when speaking or singing encode a person’s emotional intent and serve as an indication for the speakers’ interpersonal relationship in dialogs^2,3^.

Many of the current head and neck motion monitoring systems have been developed for measuring the Cervical Range of Motion (CROM) in the diagnosis of neck pain. Most of these designs employ an Inertial Measurement Unit (IMU) to obtain highly precise measurements required by the underlying application. For example, a wearable wireless inertial sensor has been proposed by Raya et al.^4^. Their design employed a MEMS-based IMU sensor consisting of the 3-axis gyroscopes, accelerometers, and magnetometers to achieve nine degrees of freedom. The measurements require the use of two of such sensors, one placed on the forehead and one on the vertebrae. Alternative methods have also been presented, Al-Nasri et al. utilized a commercially available C-stretch band to measure neck movement by placing the band around the top of the head^5^. Sarig-Bahat et al. made use of the current virtual reality technology to measure the range of motion of the neck^6^.

However, in certain non-medical situations and for our purposes, general motion monitoring instead of precise measurement of motion angles is needed. Radar technologies including Millimeter-Wave Doppler Radar and frequency-modulated continuous-wave (FMCW) radar have been utilized for this purpose in papers written by Raja et al.^7^ and Chae et al.^8^. Others like Inokuchi et al. and Chui et al. have proposed methods using cameras to provide video streaming and analyze motion using image processing^9,10^. While these methods all allow for accurate monitoring of head motion, they also present a high level of complexity in usability and design as well as low portability. Furthermore current methods present limits on usability such that the person to be monitored has to stay at a fixed location. For situations such as a classroom setting or group activity setting, where the monitoring of one’s head motion can contribute to our understanding of one’s attention, focus and emotional state, the costliness of the current designs post obstacles for such situations where a low cost head motion sensing systems are needed in bulk.

Smart thread-based sensors would provide an alternative possibility to the current head motion monitoring methods with high flexibility and efficiency. Current thread-based sensors utilize manufacturing procedures that include use of materials that directly measure strain as well as others for which strain is inferred based on changes in the electrical conductivity of an extrinsically applied coating^11–22^. With their increasing popularity, thread-based sensors have been used as means for non-obtrusive data collection methods for sweat and respiration monitoring as well as for joint and finger motion detection^23–25^. Extrinsically coated sensor thread has been used for gas detection purposes^26^ and electromagnetic devices like antennas^26–28^. Our group has developed a carbon-coated strain sensor and demonstrated its use in gait monitoring^20–22^. In this paper, we incorporate the use of thread-based sensors into the monitoring and collection of head motion. This enables a cost-effective, flexible, and portable method with high usability for everyday and large scale monitoring.

**Figure 1:**
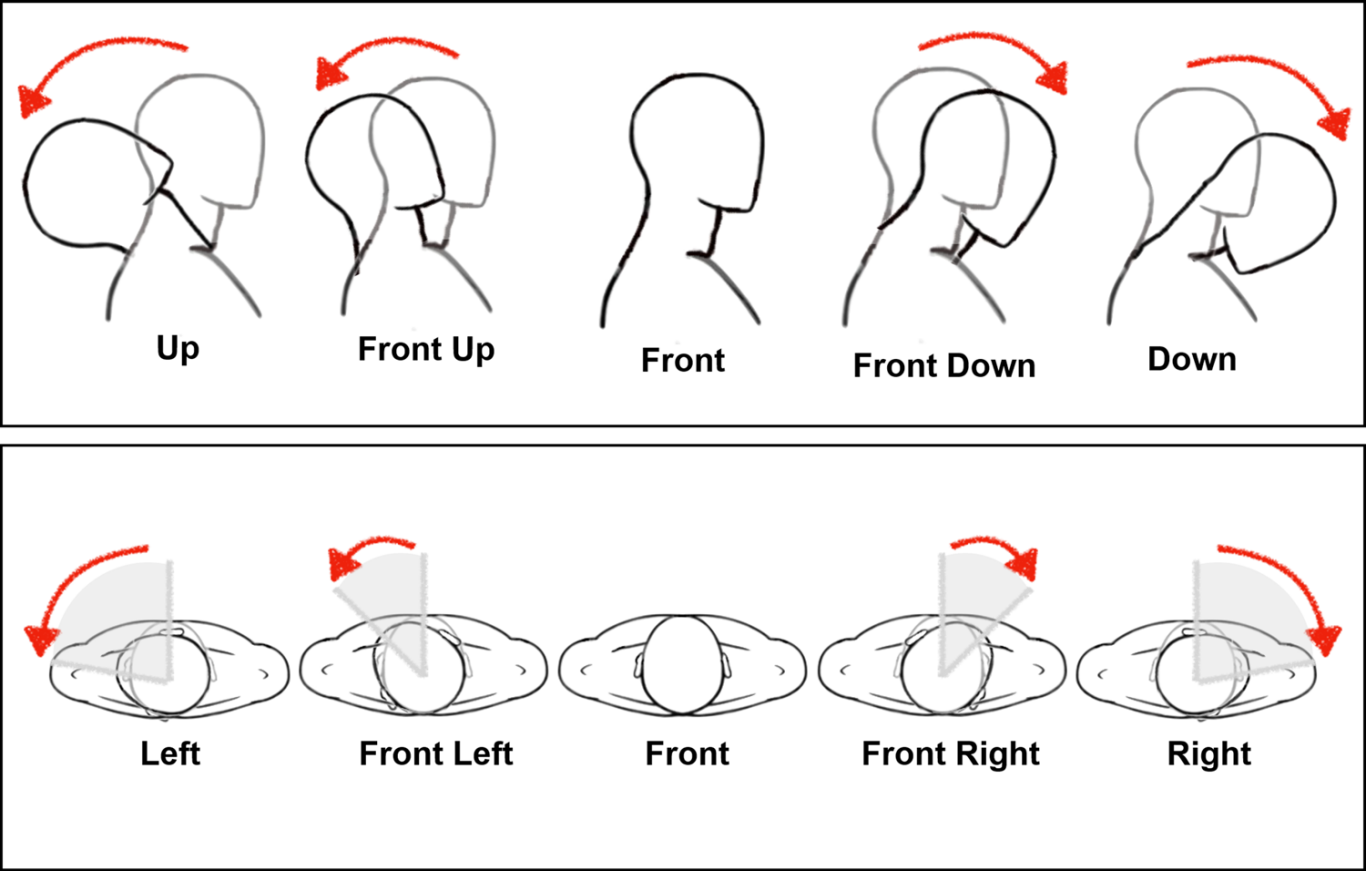
Motion classification.

We propose a method for head motion monitoring and classification using thread-based sensors. The design consists of two carbon-coated thread-based strain sensors arranged in a cross-over “X” configuration, the electronic interface for data collection, and data processing methods that allow for close to real-time determination of head orientation. The sensors are placed on the back of the neck. The sensor position is concealed thus better suited for daily use and improves from the existing c-stretch band method^5^ that places the sensor on the top of the head. Data is collected through impedance readout boards and transferred to nearby Bluetooth enabled devices. The data are divided into segments of fixed length from which a time series of features are extracted for classification of head orientation into one of nine positions. Specifically, with rotating left and right being the horizontal axis and flexing down and up being the vertical axis, the motion captured can be classified according to the location along either the horizontal or vertical axis as shown in Fig. 1. With the straightforward concepts behind the thread-based sensor, this design can be easily reproduced for other motion detection and monitoring. Moreover, the platform also exhibits a high tolerance over possible placement inaccuracy without sacrificing accuracy in classifying motions.

## Results

### Fabrication of thread sensor

**Figure 2:**
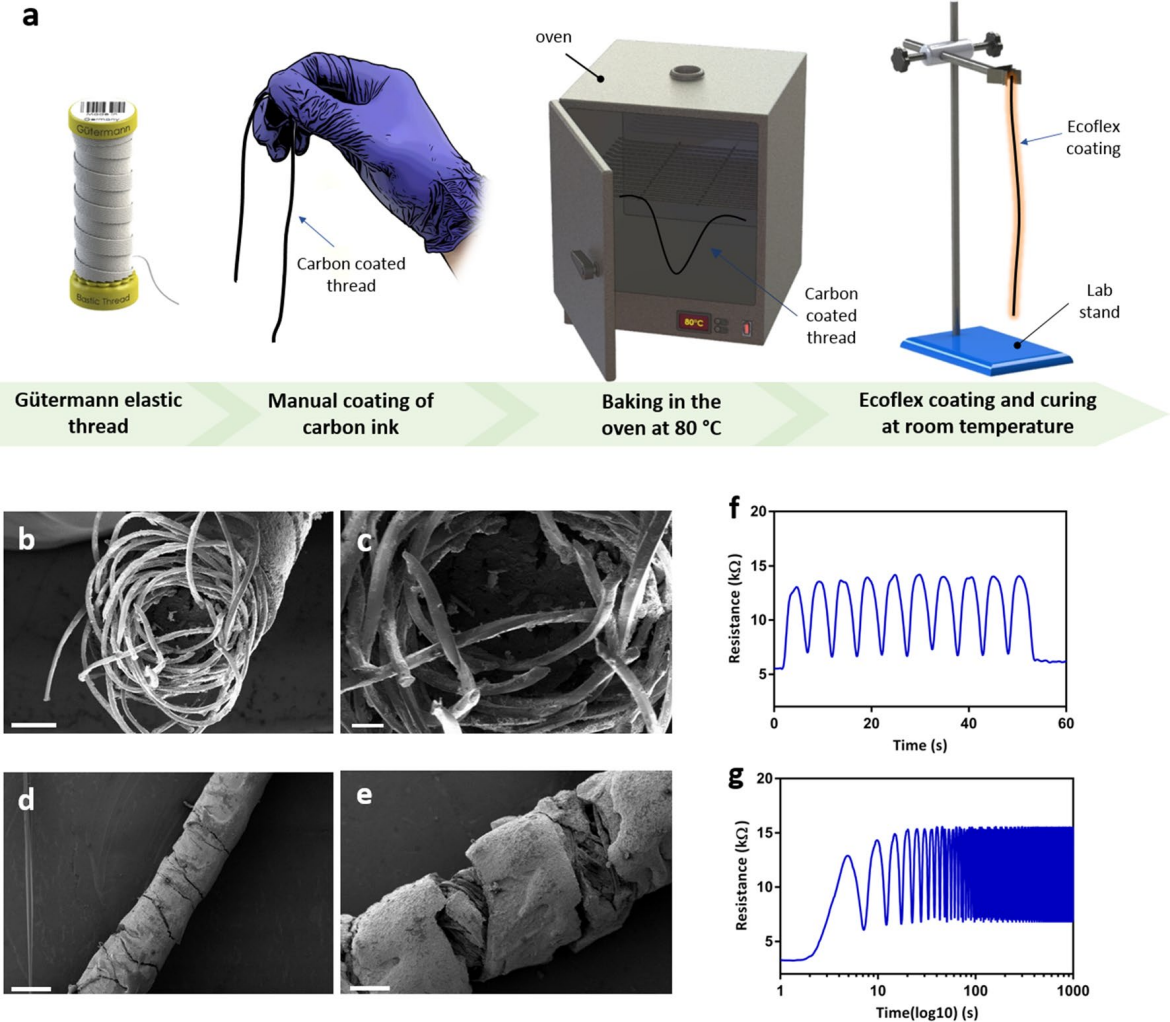
(**a**) Fabrication process of the strain sensitive thread including manual coating of carbon, baking at 80 °C and Ecoflex coating respectively (**b**) SEM image of the Gütermann elastic thread cross section with scale bar of 200 μm and (**c**) scale bar of 60 μm (**d**) SEM image of the carbon coated Gütermann elastic thread under no strain with scale bar of 500 μm (**e**) SEM image of the carbon coated Gütermann elastic thread under 10% strain with scale bar of 200 μm (**f**) dynamic cyclic test of carbon coated thread with length of 12 cm (**g**) stability test of carbon coated thread through cyclic stretching with over 2000 cycles.

The thread (Gütermann elastic thread: 64% Polyester, 36% Polyurethane; Made in Germany) is first manually coated with Carbon Resistive Ink (C-200 Carbon Resistive Ink; Applied Ink Solutions; Westborough, MA, USA). A more systematic procedure for reel-to-reel coating could also have been employed^21^. In order to ensure the thread is completely covered, the thread is first stretched to its maximum length then coated. Carbon resistive ink is manually rubbed onto the stretched thread until every portion of the thread has been covered. The thread is then transferred and baked in an oven at 80℃ for 30 min. Then, two male metal crimps are attached to the two ends of the thread by a crimping tool to enable wire connection and stable signal acquisition. As the threads are intended to be placed onto the human skin, an insulating layer is added to prevent possible signal disturbance from sensor friction on skin. A platinum-catalyzed silicone produced by Smooth-On, Inc. (Macungie, PA, USA) named EcoFlex is used to achieve this purpose. Threads are held on one end and dipped into EcoFlex and hung on one side on a rack for several seconds for the EcoFlex to be evenly coated. The EcoFlex coated threads are cured at room temperature. The fabrication process is shown in Fig. 2a. The EcoFlex coating process repeats with the other end. This provides two layers of EcoFlex which would prevent the EcoFlex layer from being torn due to excessive friction with skin in the neck movement.

### Characteristics

Using SEM images (Fig. 2b–e) the strain sensing capability of the manufactured thread sensor has been validated. The cross section of the Gütermann elastic thread shown in Fig. 2b,c demonstrates the structure inside the thread, an inflexible central core surrounded by fiber in a wound helix. When the thread is stretched, the fiber reorganizes, the helix structure increases in length prompting the central core to compress. The stretch-and-apply method employed in the manufacturing process ensures the carbon coating adheres to the surface of thread as well as the individual fiber and the central core, see Fig. 2d,e. This enables the resistance to increase when the thread experiences stretching; that is, an increase in length. This method prevents the formation of irreversible cracks on the surface of the thread. The calibration process that involves repeated motions before the beginning of data collection serves to further minimize the potential deviation caused by the development of surface cracks. The EcoFlex material used as coating is a hydrophobic and very durable polymer, this ensures no change in performance of the sensor in a humid environment caused by events like sweating. The result of a dynamic cyclic test performed is shown in Fig. 2f, the stretching of the thread corresponds to the increase in resistance values as expected. A stability test of over 2000 cycles has also been performed with the result shown in Fig. 2g. The thread demonstrated a confirmed stability over numerous cycles. The thread presents a natural drift in resistance over time and over multiple uses due to displacement of conductive carbon particulate on the thread, and as a result of plastic deformation of the EcoFlex coating. This issue can be easily accounted for in the algorithm with periodic calibration and has been investigated in our past publication^21^.

### Circuit schematics

**Figure 3:**
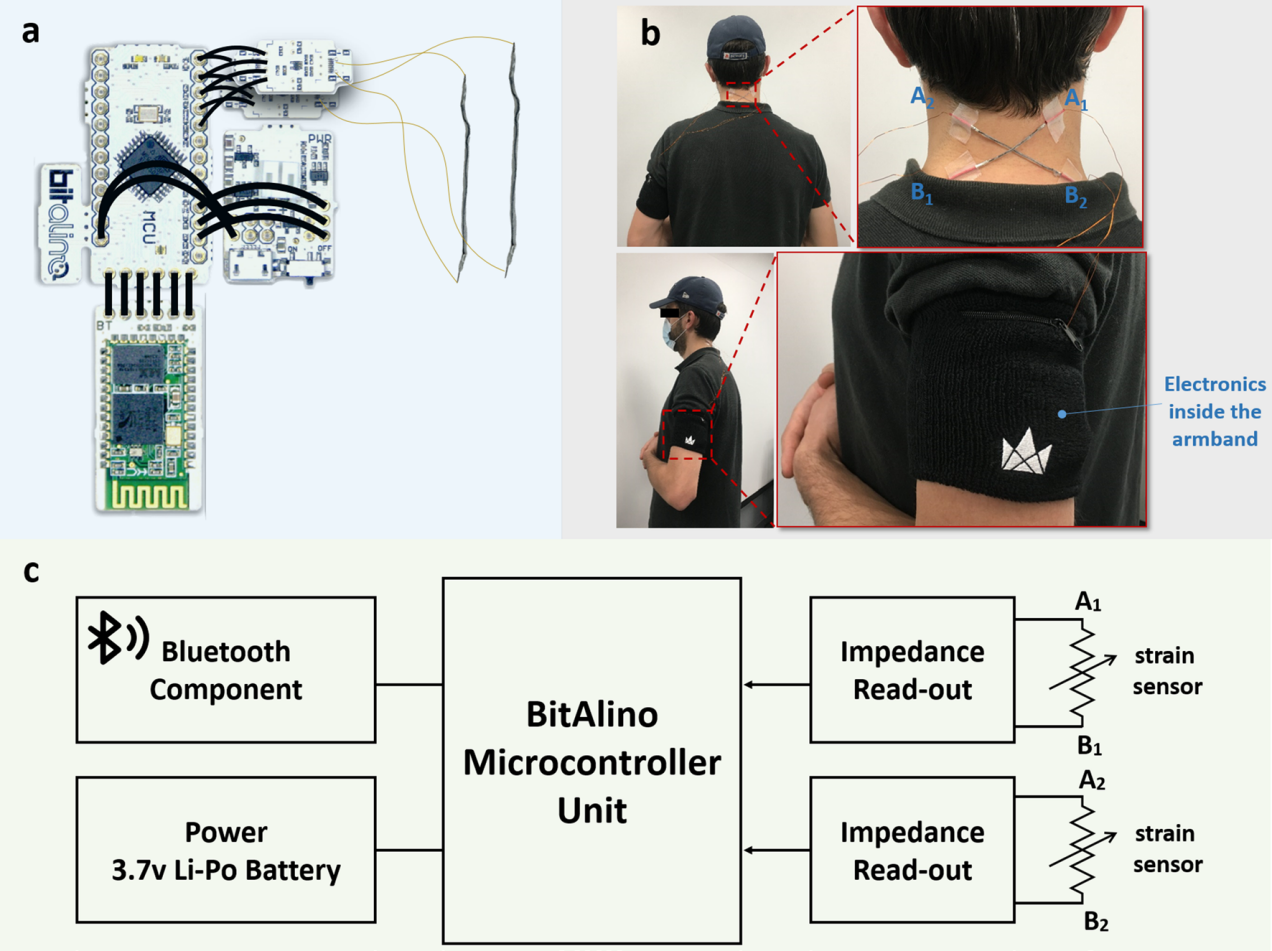
Circuit schematics and overall system. (**a**) Circuit (**b**) Thread placement on the back of the neck (**c**) Circuit diagram.

The data collection circuitry is shown in Fig. 3a,c. A microcontroller unit and two impedance read-out boards (Electrodermal Activity Sensor) designed by BITalino^29^ (Lisbon, Portugal), a toolkit developed specifically in the field of body signal collection, are used for data collection and transmission. The microcontroller board allows for real-time data streaming with a frequency of 1 kHz. The two impedance (inverse of resistance) read-out units provide the data that will be used for later processing. The two thread sensors are represented in Fig. 3c as variable resistors. The two ends of the thread sensors are soldered on flexible thin wires that are then soldered to the two data-in ports on the read-out boards. The overall circuitry is powered by a 3.7 Li-Po battery. A Bluetooth component is connected to the microcontroller unit, enabling transfer of data to nearby devices in real time. An armband, shown in Fig. 3b, containing the entire circuitry has been designed and implemented to ensure the portability of the device and is used during experiments.

### Overall system

The overall design consists of data collected from two thread sensors fabricated as described above placed on the neck. The two thread sensors are placed at the back of the neck, the position is estimated to be the location of the middle of the cervical spine region, though a precise location is not required. Two thread sensors are placed in an ‘X’ shape, one as the ‘/’ and the other as ‘\’ shown in Fig. 3b. The thread sensors are fixed on the neck by taping the two ends of the thread onto the skin using 3M Nexcare flexible clear skin adhesive tape (Saint Paul, MN, USA). The impedance values of the two thread sensors are measured and transmitted to the microcontroller unit through the BITalino impedance read-out unit. The data collected are transmitted to a nearby computer through the Bluetooth unit. The entire system is both compact and portable.

### Data processing overview

Data is collected through the thread sensors and transmitted to a nearby computer in the form of a time series of impedance values. As illustrated in Fig. S1, a processing procedure is required to convert the raw impedance values into meaningful quantities such that head orientation can be represented and identified. While we provide details of each step shortly, an overview of the approach is as follows. Before the data collection begins, each thread sensor has to be calibrated due to the differing characteristics each thread exhibits caused by nuances in the manufacturing process. After characteristic values of each sensor have been obtained, the data collection starts. The received real-time raw data are filtered to remove noise and normalized. The filtered and normalized data are then divided into overlapping data segments containing 550 data points (0.55 s). Next, a collection of features to be used as the basis for classifying head orientation are extracted from the segments. At this point, one of two paths is taken: Training and Testing. As we are using a supervised method^30^ for classifying head orientation (see Fig. 1, Introduction section), a set of training data is required. Such a library is constructed by manually assigning a label encoding the known orientation to the features for a given segment. This set of training data composed of feature vectors and their labels are used to construct an algorithm for use in the Testing Phase which, when presented with an unlabeled set of features, produces an estimate of the associated orientation. Note that for the examples in this paper, all experimentally collected data are labeled. In the training phase, a random portion of the obtained data segments will be used to generate a classifier. Using the trained classifier model, the rest of the data segments will be served as test data, the prediction made by the classifier model will be compared with the actual label as an evaluation of the success of the model.

### Calibration

**Figure 4:**
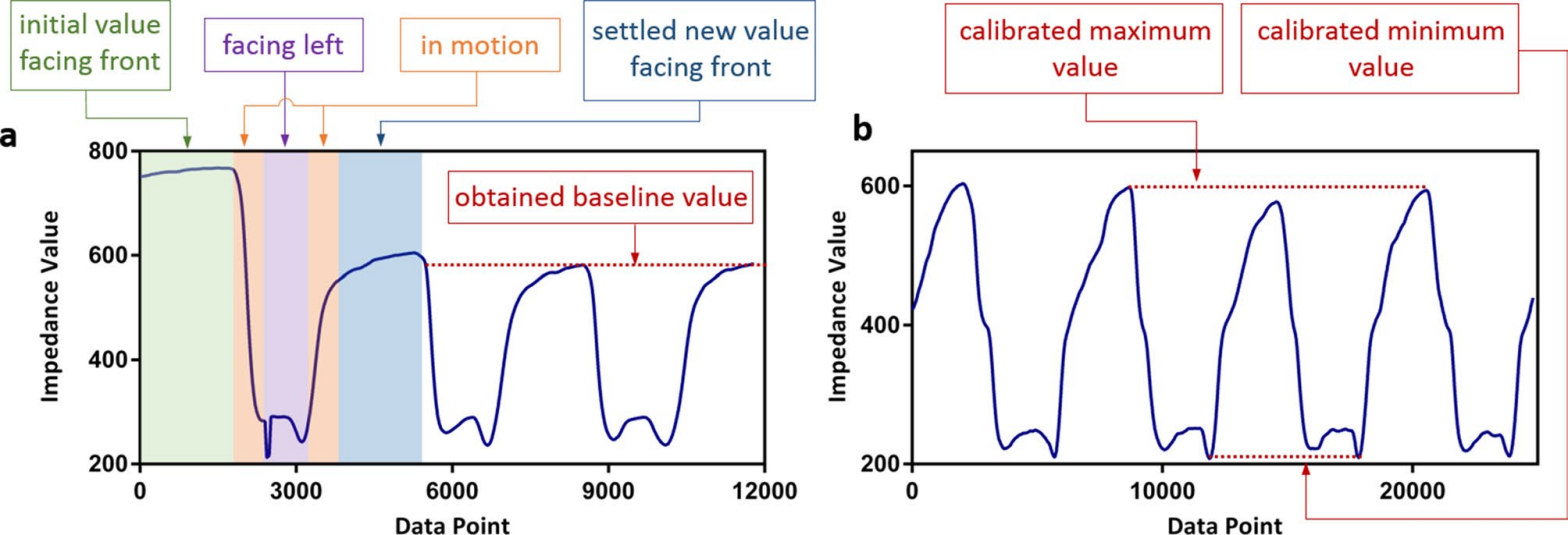
Calibration process (**a**) Calibrate for baseline value (**b**) Calibrate for maximum and minimum value.

Because the thread sensors used are manufactured manually, variations exist in the measured impedance for a given elongation. Moreover, as the threads are taped onto the skin by the two ends, the middle part of the threads are not secured onto the neck and thus exhibit spontaneous motions. Due to these conditions and characteristics, the impedance values delivered by the threads tends to fluctuate when first placed onto the neck. Thus, it is important to calibrate the sensors. Based on the objectives, three quantities are determined by our calibration process: the maximum and minimum impedance values over the full range of motions of the head as well as the settled value associated with the head in the front facing position. The value that the sensor settles at the front position is calibrated first. After the sensors are taped to the neck, the person is asked to move their head in different directions each time returning to the front-facing position. This process is repeated several times. Figure 4a demonstrates the data collected from a sample calibration process of the “/” sensor by instructing the person to repeat the rotating left motion. As shown on the plot, the leftmost values highlighted by the green box represent the initial value as the sensor is first placed on the neck while facing the front. In the calibration process, the person rotates his/her head from the front to the left and then rotates back. The rotating motion is represented in the plot by the downward and upward curves highlighted here as the orange. The pause when facing left is represented by the purple portion. As the person returns to the front position, the value of the sensor settles to a new value, represented in the plot as the blue portion. After performing the same motion several times, the settled value associated with the front position becomes clear.

In addition, a maximum and minimum impedance values associated with the full range of motion are also obtained. The sensor value exhibits the maximum change when the motion performed is deviated from the front position the most. Thus, the maximum and minimum values of the sensor are obtained by instructing the person to turn their head in each direction to the greatest extent possible. The maximum and minimum values demonstrated in the collected data from each sensor are used as the calibrated maximum and minimum values as shown in Fig. 4b.

### Filtering

**Figure 5:**
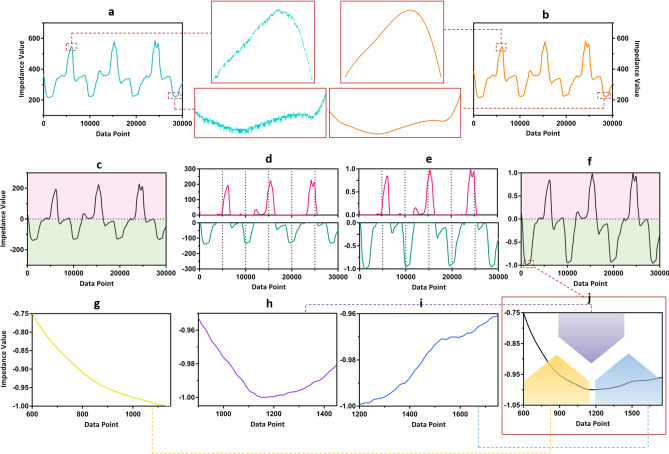
Collecting raw data, filtering, normalizing. (**a**) Raw data (**b**) Filtered data (**c**) Data shifted down by baseline value (**d**) Separating points above and below baseline value (**e**) Normalizing separately points above and below baseline value (**f**) Normalized value (**g**) Obtained data segment 1 (**h**) Obtained data segment 2 (**i**) Obtained data segment 3 (**j**) Zoomed in normalized data.

Noise in the collected data is reduced by applying a moving average filter of length 120 samples. With the sampling rate of 1000 Hz, the moving average filter acts as a low pass filter with approximately 147 Hz (0.023 rad/s) − 3 dB cutoff frequency. We determined empirically that this choice of window preserves the large scale variations of the collected data, including jumps representing the spontaneous motion of the threads, while removing the stochastic variations. Figure 5a shows the collected raw data and Fig. 5b demonstrates the filtering result from the raw data.

### Normalization

When head motion is performed, the thread sensors deviate from their original states by becoming either more stretched or more relaxed. However, from an initial relaxed position, the thread sensors tend to exhibit a greater change in impedance value when they are stretched and their length increases compared to when they relaxed and their absolute length decreases. Due to this difference, we have found it useful to normalize the data such that the changes in impedance values when associated with relaxation and elongation are better matched. Furthermore, to account for the difference between individual thread sensors, this normalization also reduces the impact of characteristic difference of individual sensors.

To achieve this goal, the data for each thread are first shifted down by the baseline value, which is the value obtained through calibration as the value associated with the front position, shown in Fig. 5c. This sets the value associated with the front position as the zero value. This also provides a more intuitive way of representing positive values as when the thread is being stretched, and negative values as when the thread becomes more relaxed. Next, as demonstrated in the pink portion of Fig. 5d,e, all positive data are scaled between 0 and 1 by treating the calibrated maximum value as 1 and the baseline value as 0. This is mathematically achieved by dividing all positive data by the calibrated maximum value. Similarly, all data below the baseline value are scaled between 0 and − 1 by treating the calibrated minimum value as − 1 and baseline value as 0. With a similar mathematical approach of dividing all negative values by − 1 multiplied by the calibrated minimum value. This process is demonstrated as the green portion in Fig. 5d,e. The final normalized data is shown in Fig. 5f. Overall, this approach to signal normalization allows any stretching and contracting to be accounted equally in the data and alleviates the effects of difference in thread characteristics.

### Data segment

Filtered and normalized incoming data are divided into overlapping segments both as the basis for feature extraction and, ultimately, to allow for close to real-time processing. Each data segment serves as the basic unit on which feature calculation is based, and orientation classification is also assigned per segment. The segment and overlap length is chosen based upon the following three criteria to ensure each window provides desired information.

1. Each window needs to be long enough to capture the key changes in the signal such as transitions from low to high values associated with intermediate positions as the head is moving such as front left, front right, etc.
2. Each window needs to encompass those relatively flat regions that are representative of extreme head positions; i.e., left, right, up and down.
3. Each window needs to have sufficient length so that it is not to be confused with the smaller variations in the signal that we hypothesize are associated with internal dynamics of the thread as it is stretched.

For the examples in this paper, each segment contains 550 data points corresponding to a time interval of 0.55 s. Adjacent segments overlap by 250 data points or 0.25 s. Figure 5g–i demonstrates three data segments obtained using this overlapping window approach for the enlarged portion of the incoming data displayed in Fig. 5j.

### Feature definitions and extraction

**Figure 6:**
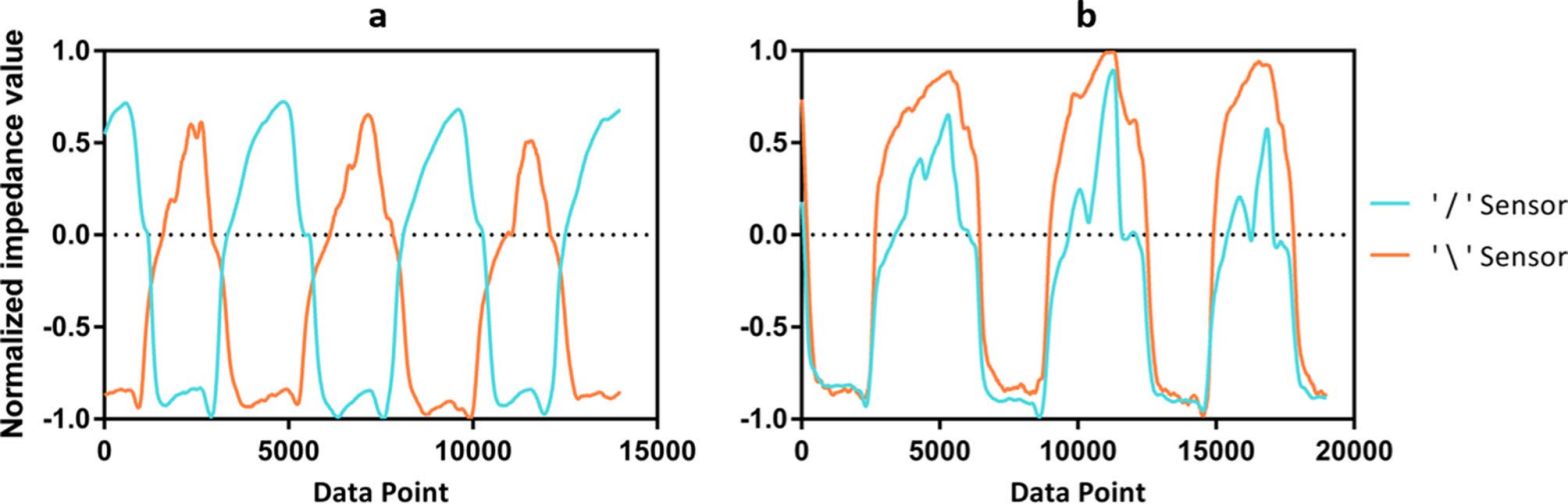
(**a**) Sample data collected from horizontal range motion (**b**) Sample data collected from vertical range motion.

From each data segment, we extract a small number of features for use in classifying head position. In this paper, we use hand-crafted features determined by the first author from visual inspection of the data. Consider the time series in Fig. 6a,b, for motions in the vertical range. Motions near the extreme positions, left, right, up, and down tend to present the most negative or positive values in both sensors, while having a flatter slope. On the other hand, motions between these extreme positions, such as front left, front right, front up, front down, tend to provide intermediate values while having a much sharper slope in the signals measured by both sensors. Given the above observation, the data segment mean and difference between minimum and maximum were used to capture these characteristics. Moreover, as shown on Fig. 6, motions in the up and down range provide highly correlated data from the two sensors, while motions in the left and right range provide inversely correlated data. Thus correlation coefficient was chosen to differentiate motions in the two axis. Several related features were also extracted based on the above observations. After extensive empirical testing, the addition of difference in mean was seen to provide a significant increase in prediction accuracy and thus was included. The final set of features we use are as follows:

Data segment mean—The mean of data points within a segment. There are two such values produced for one segment, one from each sensor.

Difference in mean—The difference between the two mean values is calculated by subtracting the segment mean from the “\” sensor from the segment mean of the “/” sensor.

Correlation Coefficient—The value is obtained by calculating the correlation coefficient between the two sets of data points for a segment produced by the two sensors. This feature measures the level of resemblance between the data produced by the two different sensors for a certain segment.

Difference between Min and Max point within a segment—The value is calculated by taking the absolute value of the maximum point minus minimum point within a segment. Compared to the correlation coefficient, which measures the strength of the relationship between the data produced by the two sensors, this feature displays the level of maximum change a data segment exhibits.

### Classifier

With the features computed and plotted, nine classifiers are trained and tested using the available data. The specifications of the classifiers used as following:

1. Linear Support Vector Machine^31^
2. Quadratic Support Vector Machine^31^
3. Cubic Support Vector Machine^31^
4. A Gaussian kernel support vector machine^31^ with a kernel scale being the square root of the number of predictors (2.4 in this case)
5. Gaussian Naive Bayes^32^
6. Kernel Naive Bayes^32^
7. Cosine KNN^33^
8. Cubic KNN^33^
9. Weighted KNN with weight being the inverse of distance squared^33^

The details of the training and testing process, referring to the flowchart in Fig. S2, are as follows. For each of 100 rounds, 75% of the generated data segments are randomly selected as training data. For each of the nine types of classifier, a classifier trained with the training data is generated. The nine generated classifiers are then tested with the remaining 25% of total segments. The testing accuracies for the models are recorded. This step is repeated 100 times. The cumulative testing result and averaged testing accuracy are recorded and analyzed.

### Experimental result

As proof of concept, a person was instructed to perform a set of motion and the predicted result from the trained classifier is compared with the actual label to demonstrate the accuracy of the system.

### Setup

The sensors are configured according to the above description. During the process of placing the thread sensor, the person is asked to hold an upright neck position. Before the start of the data collection process, calibration is performed, recording values essential to later quantification. During the data collection process, the person is asked to perform a set of motions continuously. First, the person is asked to start facing front, rotate their head to the left, return to the starting position, rotate to the right, and again return to front facing. This series of motions is repeated five times. Next, the person is asked to start facing front, tilt their head down, return to the starting position then tilt up and return to facing front. The tilting series is also performed five times. As the person is performing the instructed motions, a camera records the sequence for later labeling purposes. The collected data is divided into data segments, filtered and normalized. For each data segment, the features are calculated and stored. With reference to the video recording, each data segment is manually labeled based on the orientation classification definition.

In total, 400 data segments were generated. For each of the 100 repetitions, 300 segments were randomly selected as the training data set, and the remaining 100 as testing data. Following the training and testing procedure specified in the classifier section, the testing results were recorded.

### Data analysis

Data segment mean (µ)—In Supplementary Information Fig. S3(a-d) demonstrate the data segment mean calculated for both sensors. Comparing the result from the left and right orientation, the result from the two sensors display inverse trends as shown in Fig. S3(a) and Fig. S3(c). The “/” sensor produces the lowest mean value when the individual is facing right, with values around − 0.8 to − 1. The mean value increases as the head turns towards the left. As seen in the plot, the front right position produces a mean between −0.8 and −0.2, the front position is between −0.2 and 0.2, the front left produces values between 0.2 and 0.6, and the left position mainly in the range of 0.4 to 1. Contrastingly, the “\” sensor produces the opposite trend, the right position displays the lowest mean value of between 0.3 to 1 and the mean value decreases as the head turns towards the left. Despite the larger range exhibited by the front right position of between −0.2 to 0.2, the front position produces a mean value that is primarily between −0.2 and 0.2, consistent with the “/” sensor. The front left position produces mean values between −0.8 to −0.4, where most of the data points are distinctly below the data point cluster representing the front and front right orientations. The left position has the lowest mean values of between −1 and −0.8.

For the up and down motion range shown in Fig. S3(b) and Fig. S3(d), the two sensors demonstrate the same trend in segment mean. Both sensors associate the down position with the most negative values, which is reasonable as threads experience most stretching when the head is flexing down. As the head moves up, the mean values increase respectively. The front position again produces values around 0, this is expected as the result of normalization led the front position to be associated with zero.

**Figure 7:**
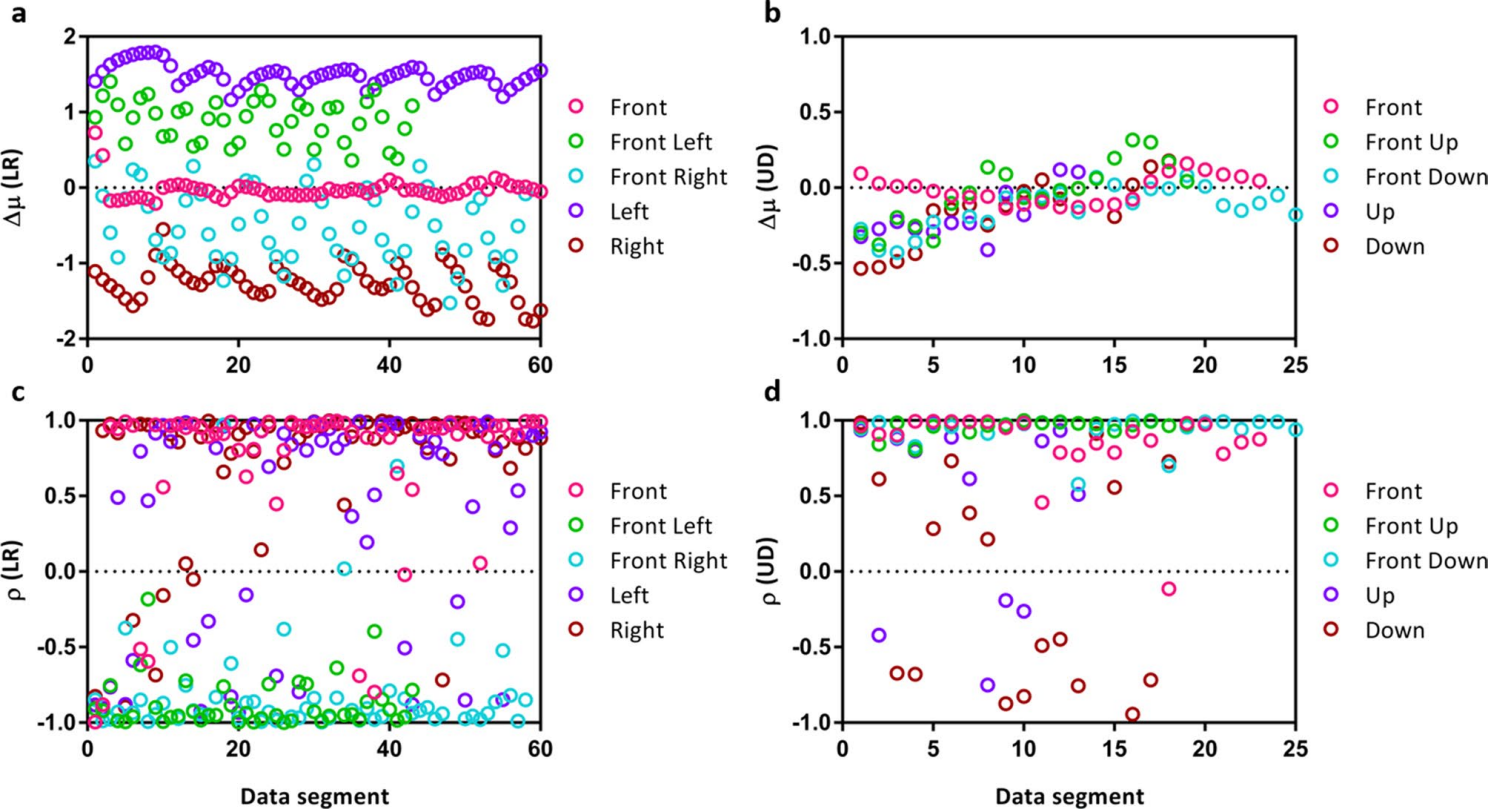
Extracted features (**a**) Difference in mean horizontal motion range (**b**) Difference in mean vertical motion range (**c**) Correlation coefficient horizontal motion range (**d**) Correlation coefficient vertical motion range.

Difference in means (Δµ)—The trends displayed in the result from segment mean are being reflected and summarized in the difference of means plots. As discussed above, the two sensors produce data that exhibits an inverse trend for left–right motion and direct trend for up-down motions. As shown in Fig. 7a, in the left and right motion range the difference of mean increases as the motion moves from right to left. For the vertical motions as shown in Fig. 7b, the differences in mean are very close to 0, as a result of high resemblance of mean values in data presented by both sensors.

Correlation Coefficient (ρ)—Examining Fig. 7c for the horizontal motion range, the sensors tend to generate negatively correlated data for the intermediate orientations, and positively correlated data for left, right, and front. This is the result of the inverse trend that is presented in the data segment mean plots, in addition to a larger change within a segment that is associated with the intermediate positions. Figure 7d demonstrates that for the vertical range, all orientations are likely to generate positively correlated data from the two sensors. This is expected as the data segment mean plot demonstrated highly correlated data produced by the two sensors.

**Figure 8:**
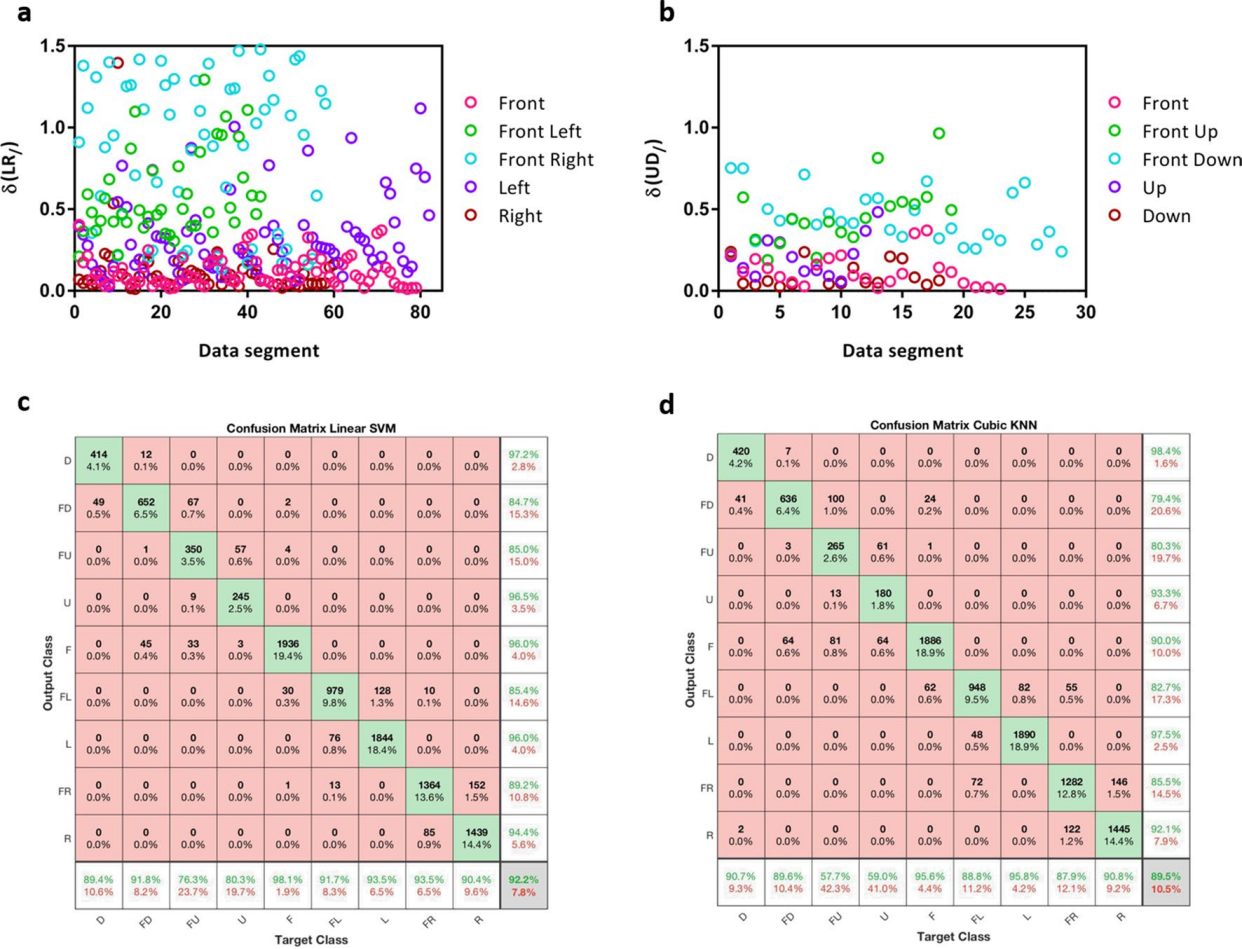
Extracted features and classifier result. (**a**) Difference between maximum and minimum value for horizontal motion range “/” sensor (**b**) Difference between maximum and minimum value for vertical motion range “/” sensor (**c**) Confusion matrix of Linear SVM testing result. (**d**) Confusion matrix of Cubic KNN testing result.

Distance between Min and Max point within a segment (δ)—Examining the plots for motions in both the horizontal and vertical range produced through the two sensors shown in Fig. 8a,b and Fig. S3(e,f), we see similar trends. Extreme and center positions including front, right, left, up and down tend to present a smaller level of change within a segment, represented here as the distances between max and min are closer to zero. In contrast, the intermediate positions such as front left, front right, front down, front up tend to present a greater level of change within a segment, as data segments classified as these motions are on the upper parts of the plot. This can be explained as the intermediate positions are associated with the action of the rotating or flexing motion of the head, resulting in a dramatic change in data produced.

Overall results—From the multiple trials of the testing process, all nine classifier models demonstrated high averaged testing accuracy of 89–92% over the 100 repetitions. Models such as Linear SVM and Gaussian kernel SVM demonstrate single round testing accuracy as high as 96%. The overall averaged testing accuracy is listed in Table 1.

**Table 1:** Averaged testing accuracy for different classifiers.

	Cosine KNN	Cubic KNN	Weighted KNN	Linear SVM	Quadratic SVM	Cubic SVM	Gaussian Kernel SVM	Kernel Naïve Bayes	Gaussian Naïve Bayes
Averaged testing accuracy	89.6%	89.5%	91.1%	92.2%	91.5%	90.5%	91.7%	90.1%	90.5%

The performance result from the best and worst models are as follows:

- Linear SVM- Linear SVM demonstrated the highest testing accuracy. Figure 8c shows the combined confusion matrix of the testing result. Most of the errors are the orientation ‘front’ confused with ‘front down’, ‘front right’ confused with ‘right’ and ‘front left’ confused with ‘left’. There are also noticeable errors where ‘front up’ is misclassified as ‘front down’.
- Cubic KNN—Cubic KNN demonstrated the lowest testing accuracy among the nine classifier models. Figure 8d shows the combined confusion matrix of the testing result. Beside from clustered errors resulting from misclassifying neighboring orientations such as ‘right’ with ‘front right’, ‘left’ with ‘front left’. This model displays a high number of misclassification between ‘front down’ and ‘front up’ as well as ‘front left’ with ‘front right’.

Overall, all nine models demonstrated high accuracies in prediction of testing data. Most of the errors are between neighboring orientations, such as front left misclassified as left or right misclassified as front right. The high testing accuracy indicates that the features selected are highly distinctive and provide strong performance over a range of classification methods.

## Discussion

A head position monitoring and classification method that demonstrates a high level of accuracy has been presented. The use of a sliding window concept to obtain data segments will ultimately allow for real-time data collection and processing, suggesting the method’s capability to process and generate results concurrently as the motions are being performed. Moreover, the design is flexible in terms of placement of the thread sensors. While the sensors are intended to be placed at the middle of the cervical spine region, slight displacement has no impact on the accuracy of the result. This was evident from the robustness of classification in spite of manual errors in placement during test and validation. The classification results demonstrate errors, if any exist, largely between neighboring orientations. In a real-life situation, when continuous segments are being collected and processed, the misclassification between neighboring orientations could be ameliorated via the development of more sophisticated, recursive tracking/filtering methods aimed at ensuring some degree of temporal “smoothness” in the estimated head orientation. The use of hand crafted features was dictated for this study by the relatively small size of the data set we had at our disposal. Future work would involve the collection of substantially more data which would then allow us to explore alternate feature generation methods such as deep nets. Different experimental conditions may well necessitate a different window length or, perhaps a more complex, adaptive approach to windowing in the event that motions occur over multiple time scales. The thread-based sensor method for head motion classification can be easily applied for motion monitoring of other parts of the body, such as elbow, knee or wrist. With a low complexity in usability and high portability, the thread-based motion monitoring method offers a new possibility of a flexible, cost-effective, and accurate design.

## Methods

The study has been reviewed and approved by Tufts University Institutional Review Board. All experiments were performed in accordance with relevant guidelines and regulations. Informed consent was obtained from all participants.

## Supplementary Information

Supplementary Information.
