# Supplementary material for: Head motion classification using thread-based sensor and machine learning algorithm
Source: Sci Rep. 2021 Jan 29;11:2646. doi: 10.1038/s41598-021-81284-7 (PMC7846730; doi:10.1038/s41598-021-81284-7)
Supplement: Supplementary file 1 — Supplementary Information. [file 41598_2021_81284_MOESM1_ESM.pdf]

Supplementary Information

Head Motion Classification Using Thread-Based Sensor and Machine Learning Algorithm

Yiwen Jiang[1], Aydin Sadeqi[1,2], Eric L. Miller[1,*], Sameer Sonkusale[1,2,*]

Scientific Reports (2020)

[1]Department of Electrical and Computer Engineering, Tufts University, Medford, MA 02155, USA

[2]Nano Lab, Department of Electrical and Computer Engineering, Tufts University, Medford, MA 02155, USA;

Corresponding Authors: Eric L. Miller (Eric.Miller@tufts.edu)
Sameer Sonkusale (Sameer.Sonkusale@tufts.edu)

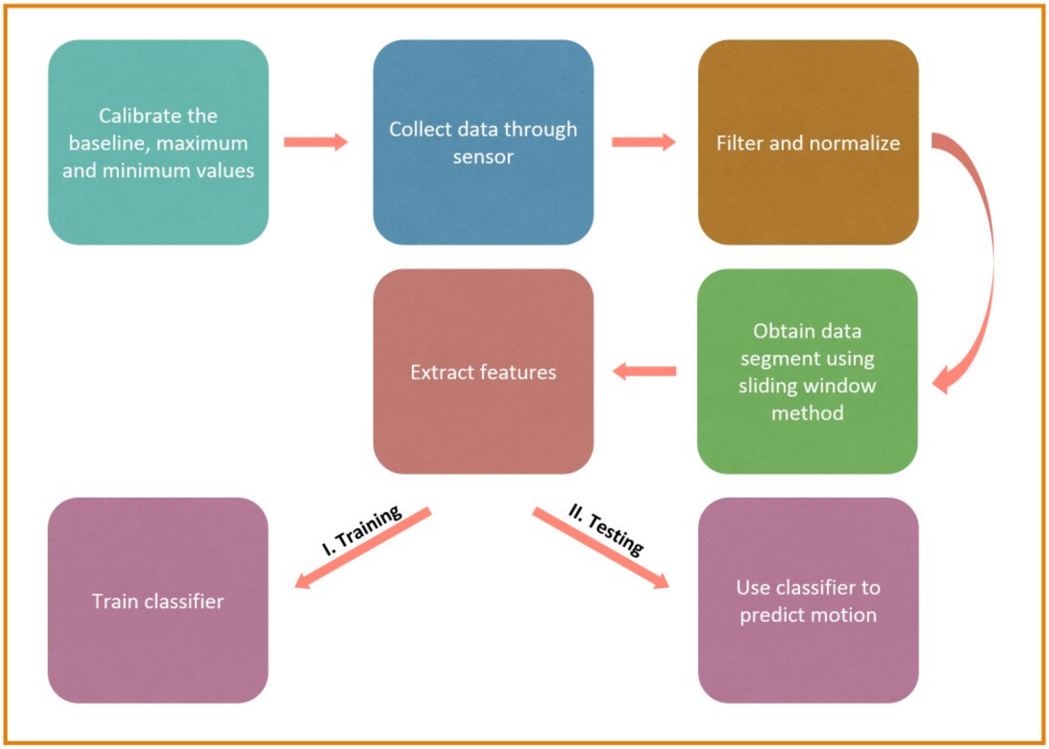

Figure S1. Flow chart of data processing.

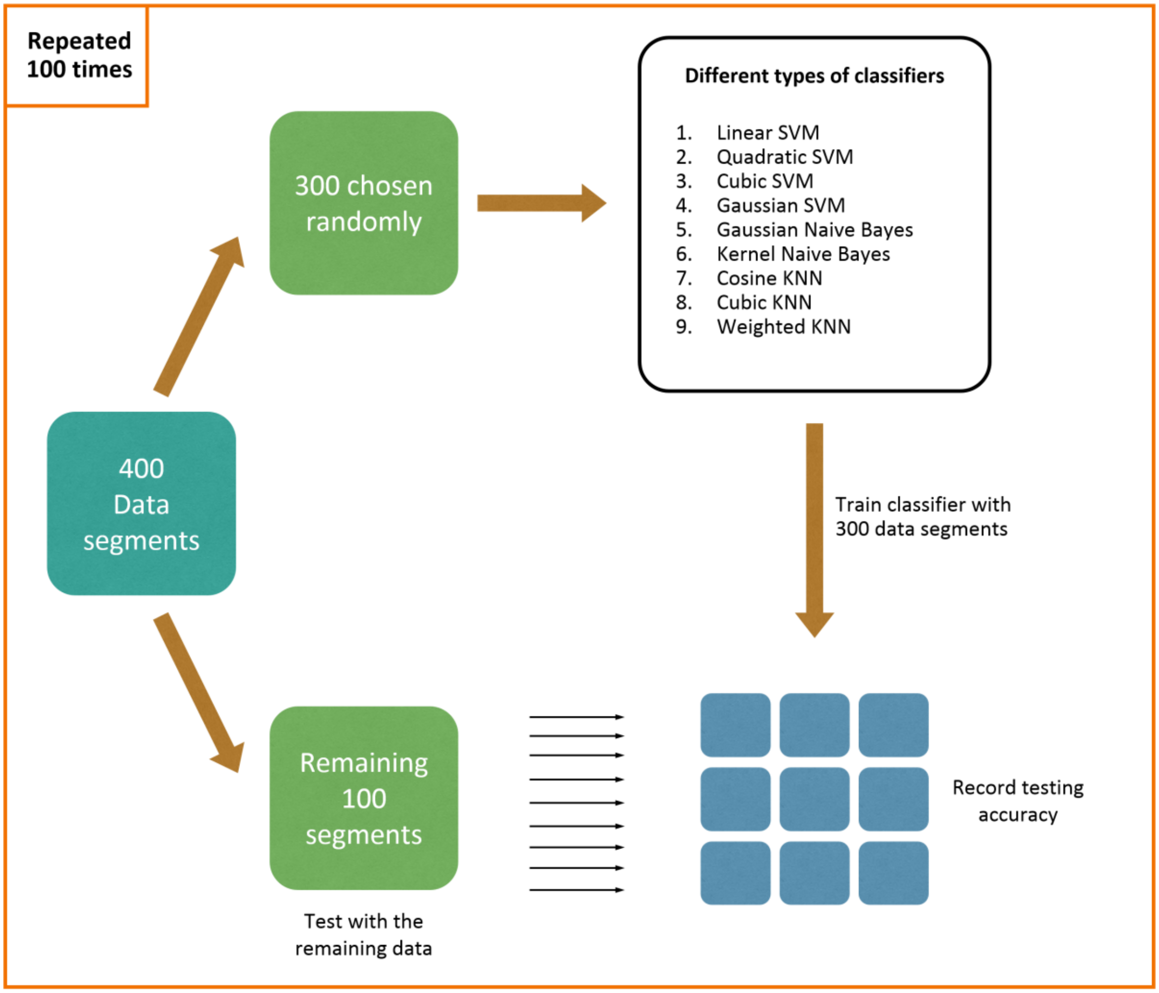

Figure S2. Flowchart of classifier testing and training process.

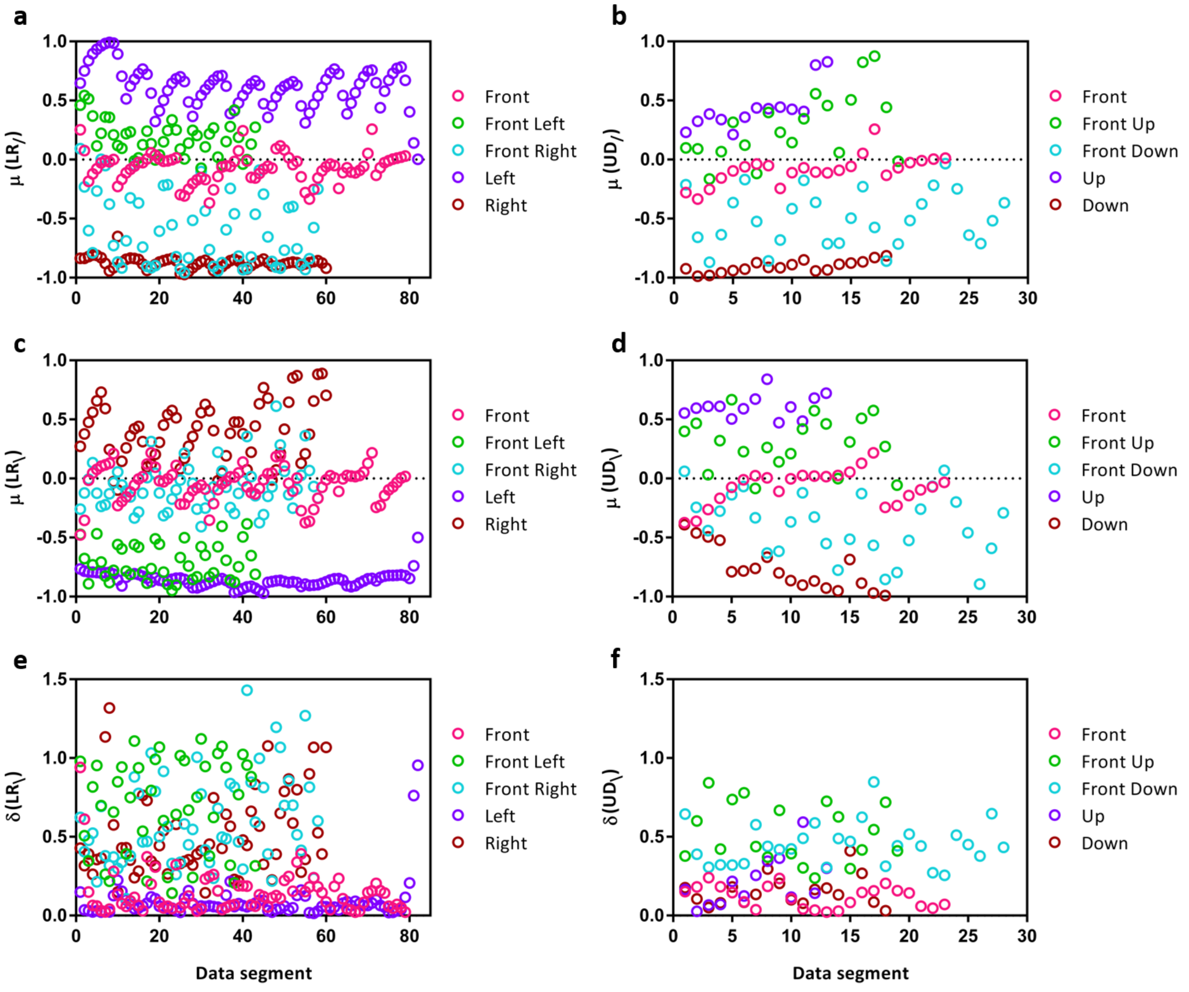

Figure S3. (a) Data segment mean horizontal motion range "/" sensor (b) Data segment mean vertical motion range "/" sensor (c) Data segment mean horizontal motion range "\" sensor (d) Data segment mean vertical motion range "\" sensor (e) Difference between maximum and minimum value for horizontal motion range "\" sensor (f) Difference between maximum and minimum value for vertical motion range "\" sensor.
